# IL-21 Promotes CD4 T Cell Responses by Phosphatidylinositol 3-Kinase–Dependent Upregulation of CD86 on B Cells

**DOI:** 10.4049/jimmunol.1302082

**Published:** 2014-01-27

**Authors:** Kesley Attridge, Rupert Kenefeck, Lukasz Wardzinski, Omar S. Qureshi, Chun Jing Wang, Claire Manzotti, Klaus Okkenhaug, Lucy S. K. Walker

**Affiliations:** * Medical Research Council Centre for Immune Regulation, University of Birmingham Medical School, Birmingham B15 2TT, United Kingdom;; † Institute of Immunity and Transplantation, University College London Medical School, London NW3 2PF, United Kingdom; and; ‡ Laboratory of Lymphocyte Signalling and Development, Babraham Institute, Cambridge CB22 3AT, United Kingdom

## Abstract

The cytokine IL-21 is a potent immune modulator with diverse mechanisms of action on multiple cell types. IL-21 is in clinical use to promote tumor rejection and is an emerging target for neutralization in the setting of autoimmunity. Despite its clinical potential, the biological actions of IL-21 are not yet fully understood and the full range of effects of this pleiotropic cytokine are still being uncovered. In this study, we identify a novel role for IL-21 as an inducer of the costimulatory ligand CD86 on B lymphocytes. CD86 provides critical signals through T cell–expressed CD28 that promote T cell activation in response to Ag engagement. Expression levels of CD86 are tightly regulated in vivo, being actively decreased by regulatory T cells and increased in response to pathogen-derived signals. In this study, we demonstrate that IL-21 can trigger potent and sustained CD86 upregulation through a STAT3 and PI3K-dependent mechanism. We show that elevated CD86 expression has functional consequences for the magnitude of CD4 T cell responses both in vitro and in vivo. These data pinpoint CD86 upregulation as an additional mechanism by which IL-21 can elicit immunomodulatory effects.

## Introduction

Interleukin-21 is known to influence multiple parameters of the immune response. The clinical importance of this pathway was first appreciated nearly a decade ago with the demonstration that IL-21 could augment antitumor immunity (1, 2), and this has since become an active area of research (3–5). In addition to augmenting immunity against tumors, IL-21 signaling can directly induce apoptotic pathways in chronic lymphocytic leukemia (CLL) B cells (6, 7) and diffuse large B cell lymphoma (8).

The role of IL-21 in T cell–dependent B cell responses has been extensively documented. IL-21 critically regulates Ab production, partly in cooperation with IL-4 (9), and it promotes plasma cell differentiation in both mice (10) and humans (11). The intimate interaction between follicular helper T (T_FH_) cells and germinal center B cells is also shaped by provision of IL-21; T_FH_ cell–derived IL-21 directly targets germinal center B cells, reinforcing their fate decision by sustaining bcl6 expression (12, 13).

Alongside effects on B cells, several studies have also reported that IL-21 promotes T cell activation. Pre-exposure to IL-21 has been shown to increase the Ag responsiveness of CD8 T cells (14) and permit triggering by weak TCR agonists (15). CD4 T cell responses can also be augmented by IL-21, in part due to its ability to counteract regulatory T cell suppression (16, 17). The mechanisms by which IL-21 directly or indirectly promotes T cell responses are not yet fully defined. In this study we identify a novel role for IL-21 in upregulating the expression of the costimulatory ligand CD86 on B cells. We show that this requires activation of the PI3K pathway and is dependent on the PI3K subunit p110δ, a molecule currently being targeted in the setting of several B cell malignancies (CLL, non-Hodgkin lymphoma) (18). The increased expression of CD86 on B cells is shown to have functional consequences for T cell expansion both in vitro and in vivo. Collectively, these data suggest an additional mechanism by which IL-21 may augment adaptive immune responses and reveal a further level of T cell/B cell interaction directed by this cytokine.

## Materials and Methods

### Mice

DO11.10 TCR transgenic and BALB/c mice were purchased from The Jackson Laboratory. IL-21R^−/−^ mice were provided by Manfred Kopf (ETH Zurich) and were bred with DO11.10 TCR transgenic mice to generate IL-21R^−/−^ DO11.10 TCR transgenic progeny. p110δ^D910A^ mice were provided by K.O. Mice were housed at the University of Birmingham Biomedical Services Unit or at the University College London and used according to Home Office and institutional guidelines.

### Flow cytometry

Cells were stained with mAbs against CD25 (PC61.5; eBioscience), CD4 (LT34; eBioscience), CD19 (1D3), CD86 (GL1; eBioscience), CD80 (16-10A1), pSTAT1 (14/P-STAT1), pSTAT3 (49/P-STAT3), pSTAT5 (clone 47), and DO11.10 TCR (KJ1.26; eBioscience). All Abs were purchased from BD Biosciences unless otherwise indicated. For pSTAT staining, cells were fixed in 4% paraformaldehyde for 10 min and permeabilized with 100% ice-cold methanol for 30 min. Statistics were performed using an unpaired two-tailed *t* test with a 95% confidence interval.

### Short-term splenocyte cultures

BALB/c splenocytes (1 × 10^5^) were cultured for 15–16 h alone, with IL-21 at 25, 50, 100 or 200 ng/ml (PeproTech), or with 1 μg/ml LPS (Sigma-Aldrich). For time course experiments, cells were harvested at 2, 4, 6, 8, or 15 h.

### Short-term B cell cultures

Magnetic separation (Miltenyi Biotec) was used to purify CD19^+^ B cells from BALB/c or p110δ^D910A^ spleen. Cells (1 × 10^6^) were cultured for 16 h alone or in the presence of 200 ng/ml IL-21 (PeproTech) or 10 ng/ml IL-4 (PeproTech). For STAT3 inhibition experiments cultures were supplemented with 10, 50, or 100 μM S3I-201 (Calbiochem) as indicated. For PI3K inhibition experiments cultures were supplemented with 10 μM LY-294002 (Invitrogen) as indicated. For assessment of activated STAT proteins cells were cultured for 2 h alone or in the presence of 200 ng/ml IL-21 (PeproTech). For experiments to determine the target of IL-21, 2.5 × 10^4^ BALB/c or IL-21R^−/−^ B cells were cultured with 2.5 × 10^4^ magnetically separated (Miltenyi Biotec) CD4^+^CD25^−^ T cells from BALB/c or IL-21R^−/−^ lymph node for 16 h alone or in the presence of 200 ng/ml IL-21 (PeproTech).

### Confocal microscopy

Magnetic separation (Miltenyi Biotec) was used to purify CD19^+^ B cells from BALB/c spleen. Cells (1 × 10^6^) were cultured for 6 h alone, with 200 ng/ml IL-21 (Peprotech), with 10 μg/ml cycloheximide (Sigma-Aldrich), or with both. Cells were stained with mAb against CD86 (GL1; eBioscience) and imaged using glass bottom culture dishes (MatTek). Imaging was carried out using a ×100 oil immersion objective.

### RT-PCR

Magnetic separation (Miltenyi Biotec) was used to purify CD19^+^ B cells from BALB/c spleen. mRNA was isolated at this point or after culture of 1 × 10^6^ cells for 16 h alone or in the presence of 200 ng/ml IL-21 (PeproTech). Quantitative PCR was performed to assess the expression of β_2_-microglobulin (Eurofins MWG Operon) or CD86 (TaqMan gene expression assay; Applied Biosystems).

### In vitro proliferation assays

Magnetic separation (Miltenyi Biotec) was used to purify CD4^+^CD25^−^ T cells from IL-21R^−/−^ lymph node. Cells (2.5 × 10^4^) were cultured with 2.5 × 10^4^ CD19^+^ B cells from BALB/c or IL-21R^−/−^ spleen, with 0.8 μg/ml anti-CD3 (BD Biosciences) alone or in the presence of 10 μg/ml anti-CD86 (Bio X Cell). Triplicate wells were pooled and harvested at day 1 to assess CD86 expression or day 3 to determine cell counts by flow cytometry.

### Adoptive transfers

CD19^+^ B cells from BALB/c or IL-21R^−/−^ spleen were isolated by magnetic separation (Miltenyi Biotec) and cultured for 16 h with 1 μg/ml OVA peptide. Peptide-pulsed cells (3 × 10^6^) were injected i.v. into IL-21R^−/−^ recipients. Magnetic separation (Miltenyi Biotec) was used to purify CD4^+^ cells from IL-21R^−/−^ DO11.10 TCR^+^ lymph node. Twenty-four hours after B cell transfer, a total of 2 × 10^6^ CellTrace Violet–labeled IL-21R^−/−^ DO11.10 TCR^+^ T cells were injected i.v. and 1 μg IL-21 (PeproTech) or PBS i.p. Where indicated recipients were also given 100 μg anti-CD86 (Bio X Cell) or PBS i.p. immediately after T cell transfer. Mice were culled at day 7 for analysis of inguinal lymph node and splenic cells.

## Results

### IL-21 upregulates CD86 expression on B cells

**FIGURE 1.**
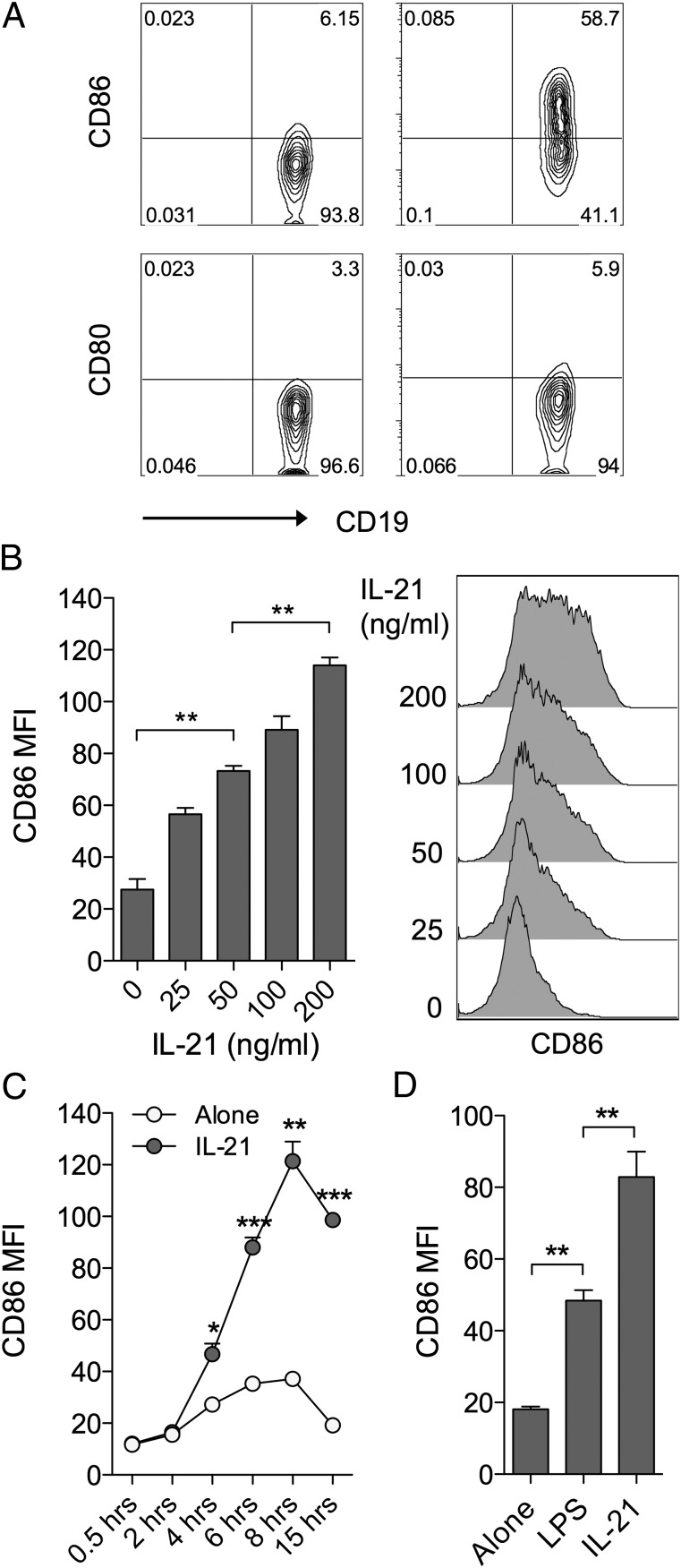
IL-21 upregulates CD86 expression on B cells. (**A**) BALB/c splenocytes (1 × 10^5^) were cultured for 16 h alone or in the presence of 200 ng/ml IL-21. Plots show representative CD80 and CD86 expression for gated CD19^+^ B cells. (**B**) BALB/c splenocytes (1 × 10^5^) were cultured for 16 h alone or in the presence of the indicated doses of IL-21. Histograms show CD86 expression for gated CD19^+^ B cells and graph shows collated CD86 mean fluorescence intensity (MFI) data. (**C**) BALB/c splenocytes (1 × 10^5^) were cultured alone or in the presence of 200 ng/ml IL-21 and harvested at the indicated time points. Graph shows collated CD86 MFI data for gated CD19^+^ B cells. (**D**) BALB/c splenocytes (1 × 10^5^) were cultured for 16 h alone or in the presence of either 200 ng/ml IL-21 or 1 μg/ml LPS. Graph shows collated CD86 MFI data for gated CD19^+^ B cells. Data are representative of at least three independent experiments. *p < 0.05, **p < 0.01, ***p < 0.001.

To explore the influence of IL-21 on costimulatory ligand expression, murine splenocytes were cultured in the presence or absence of IL-21 for 16 h and the expression of CD86 and CD80 was assessed by flow cytometry. We noted a marked elevation of CD86, but not CD80, on CD19^+^ B cells in the timeframe examined (Fig. 1A). Titration (Fig. 1B) and time course (Fig. 1C) data established that IL-21–mediated CD86 upregulation was dose-dependent and appeared maximal at ∼8 h following stimulation. The magnitude of CD86 upregulation observed in response to IL-21 was comparable, if not greater, than that seen with a 1 μg/ml dose of LPS, a TLR ligand known to drive CD86 upregulation (Fig. 1D). In contrast, splenic dendritic cells (DCs) showed some CD86 expression at baseline and this was not further elevated by IL-21 under the conditions assessed (Supplemental Fig. 1).

### CD86 upregulation requires direct signaling to B cells and protein neosynthesis

**FIGURE 2.**
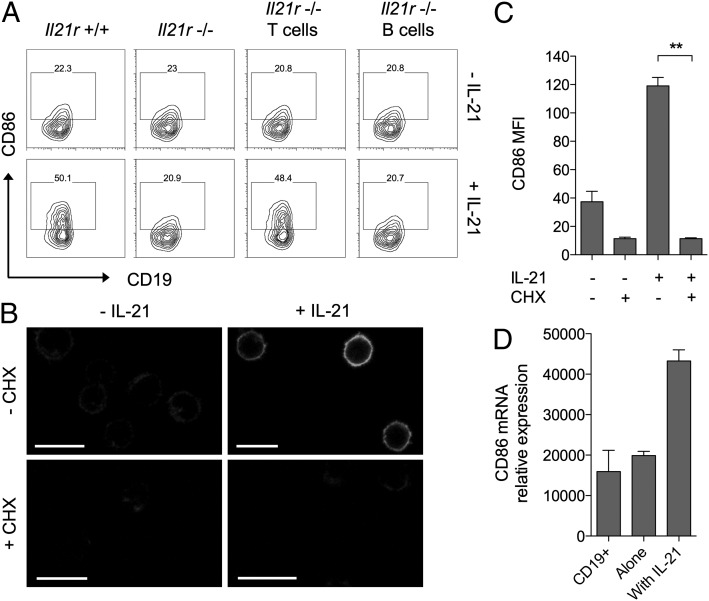
Induction of CD86 by IL-21 requires direct signaling to B cells and is dependent on protein neosynthesis. (**A**) BALB/c CD4^+^CD25^−^ conventional T cells (2.5 × 10^4^) were cultured with 2.5 × 10^4^ CD19^+^ B cells for 16 h alone or in the presence of 200 ng/ml IL-21. Cell populations were deficient for the IL-21R as indicated. Plots show CD86 expression for gated CD19^+^ B cells. (**B**) BALB/c CD19^+^ B cells (1 × 10^6^) were cultured for 6 h alone, with 200 ng/ml IL-21, with 10 μg/ml cycloheximide, or both. Representative confocal microscopy images show CD86 staining. Scale bars, 10 μm. (**C**) Graph shows collated CD86 mean fluorescence intensity (MFI) for cells from (B) analyzed by flow cytometry. Data are representative of three independent experiments. (**D**) BALB/c CD19^+^ B cells (1 × 10^6^) were cultured for 16 h alone or in the presence of 200 ng/ml IL-21. Graph shows relative CD86 mRNA expression for cultured cells and freshly isolated CD19^+^ B cells. Data are representative of three independent experiments. **p < 0.01.

Because the above assays did not use purified B cells, it remained possible that IL-21 was altering CD86 expression indirectly by acting on a different cell type. In this regard we have previously shown that conventional CD4 T cells express high levels of IL-21R and can respond to IL-21 by acquiring resistance to regulatory T cell suppression (19). To dissect whether regulation of CD86 expression involved direct signaling to B cells or was an indirect consequence of IL-21 signaling to T cells, we took advantage of mice genetically deficient for the IL-21R (20). Cocultures of T cells and B cells were established in which the cells derived from either wild-type or IL-21R–deficient mice and these were incubated for 16 h with or without IL-21. As expected, when both T and B cells expressed IL-21R, IL-21 upregulated CD86 expression (Fig. 2A, *far left panels*) and when neither cell type expressed IL-21R, CD86 was unchanged (Fig. 2A, *second panels*). Crucially, in situations where B cells were IL-21R^+/+^ but T cells were IL-21R^−/−^, addition of IL-21 retained the capacity to upregulate CD86 (Fig. 2A, *third panels*). Furthermore, preventing B cells alone from receiving IL-21 signals completely abrogated the ability of IL-21 to upregulate CD86 (Fig. 2A, *far right panels*). Collectively, these data indicate that direct signaling of IL-21 to B cells is responsible for mediating CD86 upregulation.

The upregulation of CD86 by the cytokine IFN-γ has been reported to be independent of protein neosynthesis (21). To examine whether upregulation of CD86 by IL-21 required protein neosynthesis, we performed experiments using purified B cells in the presence or absence of cycloheximide. The ability of IL-21 to upregulate B cell CD86 expression was completely abrogated in the presence of cycloheximide, indicating a requirement for protein neosynthesis (Fig. 2B, 2C). We next assessed whether upregulation of CD86 by IL-21 was transcriptionally regulated; this revealed that mRNA for CD86 was strongly upregulated by exposure to IL-21 (Fig. 2D). Taken together, these data indicate that de novo transcription and translation of CD86 mRNA is required for IL-21–mediated CD86 upregulation.

### CD86 upregulation requires STAT3 and PI3K

**FIGURE 3.**
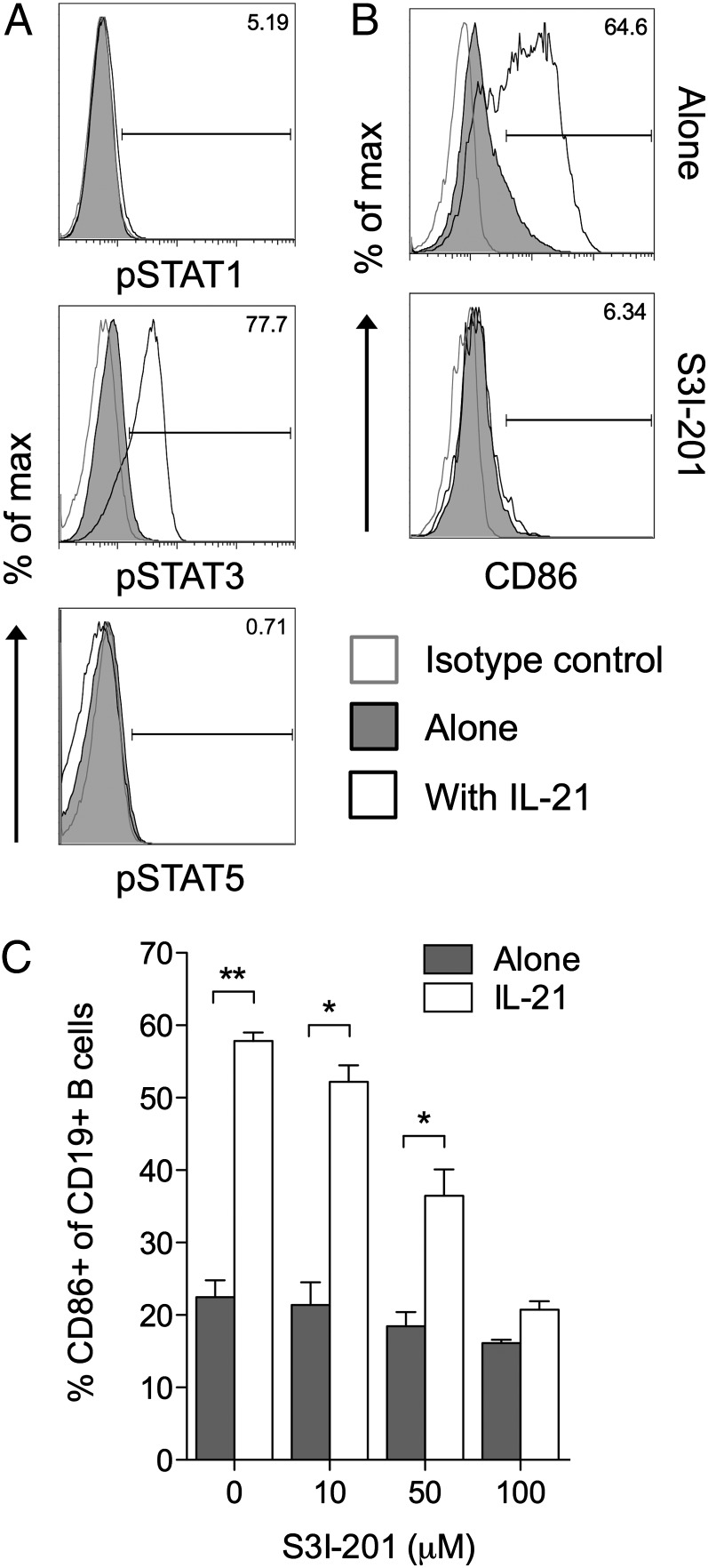
Induction of CD86 by IL-21 is dependent on STAT3 activation. (**A**) BALB/c CD19^+^ B cells (1 × 10^6^) were cultured for 2 h alone or in the presence of 200 ng/ml IL-21. Histograms show representative staining for phosphorylated STAT proteins as indicated. (**B**) BALB/c CD19^+^ B cells (1 × 10^6^) were cultured for 16 h alone or in the presence of 200 ng/ml IL-21, 100 μM S3I-201, or both. Histograms show representative CD86 expression for gated CD19^+^ B cells. (**C**) Graph shows collated CD86 expression data for cells from B when cultured with the indicated doses of S3I-201. Data are representative of three independent experiments. *p < 0.05, **p < 0.01.

IL-21 signaling is known to involve STAT3 activation in both mouse (22) and human (23, 24) B cells, and the ability of STAT3 to mediate IL-21 effects has been demonstrated using B cells isolated from STAT3-deficient patients (25). We confirmed the ability of IL-21 to activate the STAT3 pathway in B cells (Fig. 3A) and then sought to determine whether this pathway was involved in IL-21–mediated CD86 upregulation. In the presence of the STAT3 inhibitor S3I-201, IL-21 was no longer able to induce significant CD86 upregulation (Fig. 3B, 3C), suggesting a critical role for STAT3 in this process.

**FIGURE 4.**
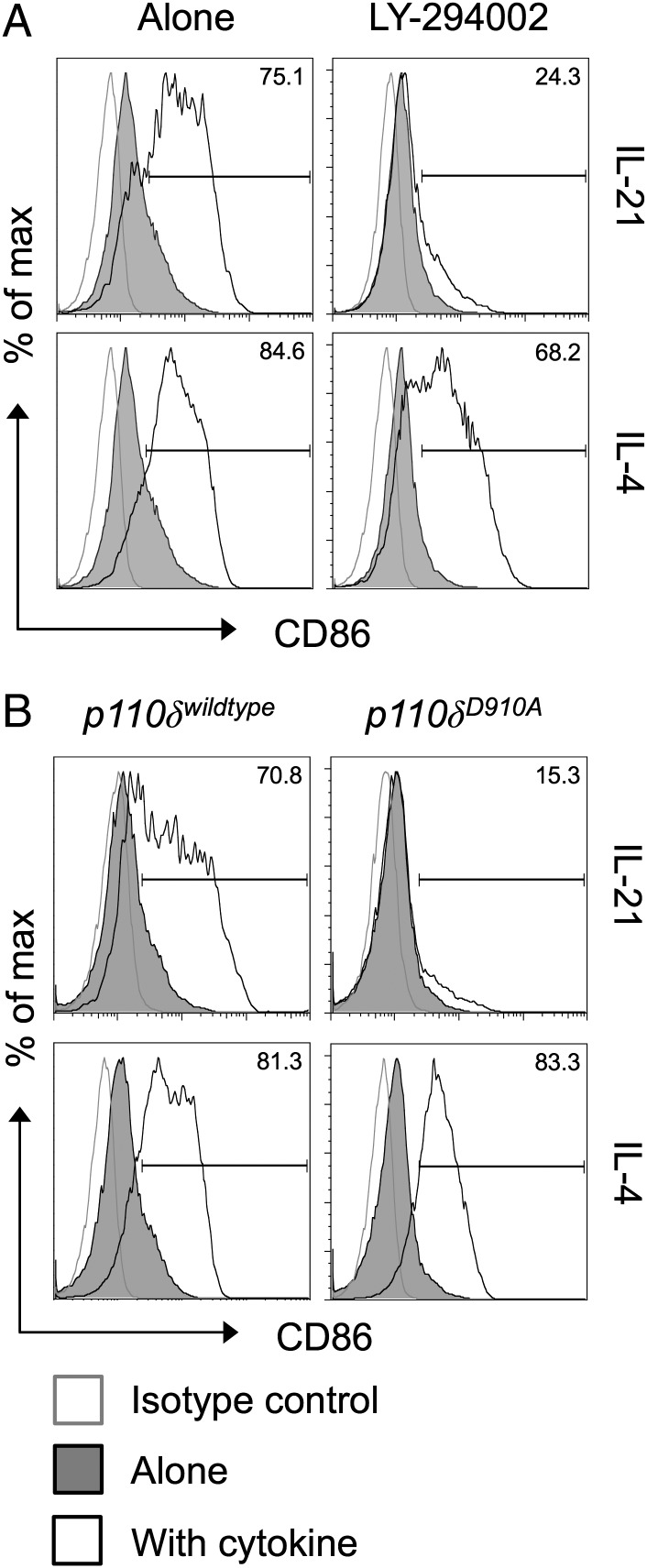
Induction of CD86 by IL-21 is dependent on PI3K p110δ. (**A**) BALB/c CD19^+^ B cells (1 × 10^6^) were cultured for 16 h alone or in the presence of 200 ng/ml IL-21 or 10 ng/ml IL-4. Where indicated, cultures also contained 10 μM LY-294002. Histograms show CD86 expression for gated CD19^+^ B cells. (**B**) BALB/c CD19^+^ B cells (1 × 10^6^) were cultured for 16 h alone or in the presence of 200 ng/ml IL-21 or 10 ng/ml IL-4. Cells expressed the wild-type or mutant isoforms of PI3K P110δ as indicated. Histograms show CD86 expression for gated CD19^+^ B cells. Data are representative of three independent experiments.

A second major pathway triggered by IL-21 is the activation of the enzyme PI3K (22, 26), and the ability of BCR engagement to upregulate CD86 is known to be dependent on PI3K signaling (27). To determine whether IL-21 upregulates CD86 via a PI3K-dependent pathway, we first employed the PI3K inhibitor LY-294002. Strikingly, IL-21–mediated CD86 upregulation was completely abrogated in the presence of this inhibitor (Fig. 4A). The related cytokine IL-4 retained the ability to upregulate CD86 in the presence of the PI3K inhibitor, consistent with previous studies (27). We next took advantage of mice expressing a catalytically inactive form of the PI3K p110δ subunit (D910A mice) (28), because this subunit is known to be important in the B cell lineage (29, 30). Purified B cells from D910A animals failed to upregulate CD86 in response to IL-21, whereas their ability to upregulate CD86 in response to IL-4 remained intact (Fig. 4B). Collectively, these data indicate that IL-21 uses STAT3 and PI3K to drive CD86 upregulation and pinpoint p110δ as the critical PI3K subunit responsible.

### IL-21–mediated CD86 upregulation has functional consequences in vitro

**FIGURE 5.**
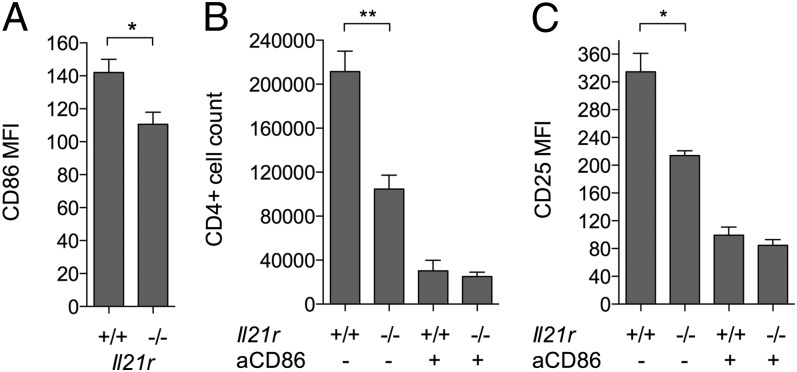
IL-21 signaling to B cells promotes CD86-dependent T cell proliferation. IL-21R^−/−^ CD4^+^CD25^−^ conventional T cells (2.5 × 10^4^) were cultured with 0.8 μg/ml anti-CD3 and 2.5 × 10^4^ IL-21R^−/−^ or IL-21R^+/+^ CD19^+^ B cells alone or in the presence of 10 μg/ml anti-CD86. (**A**) Graph shows collated CD86 mean fluorescence intensity (MFI) data for gated CD19^+^ B cells harvested after 16 h in the absence of anti-CD86 blocking Ab. (**B**) Graph shows collated data for absolute CD4^+^ conventional T cell counts after harvest at day 3 when cultured with IL-21R^−/−^ or IL-21R^+/+^ B cells as indicated. (**C**) Graph shows collated CD25 MFI data for CD4^+^ conventional T cells from (B). *p < 0.05, **p < 0.01.

To test whether the upregulation of CD86 in response to IL-21 had functional consequences, in vitro T cell proliferation assays were performed. CD4^+^CD25^−^ T cells from IL-21R^−/−^ mice were used to preclude direct effects of IL-21 on the T cells themselves. IL-21R^−/−^ T cells were activated by anti-CD3 in the presence of B cells that were either wild-type or IL-21R deficient; in this setting any IL-21 made by the T cells would potentially be able to upregulate CD86 on the wild-type B cells but not the IL-21R^−/−^ ones. In line with this, CD86 was upregulated to a greater extent on the IL-21R^+/+^ B cells than on the IL-21R^−/−^ ones (Fig. 5A). IL-4 did not appear to contribute to CD86 upregulation in this setting as assessed by inclusion of blocking anti–IL-4 Ab (data not shown). The increased CD86 expression on IL-21R^+/+^ B cells was associated with significantly greater T cell proliferation (Fig. 5B) and activation marker expression (Fig. 5C and data not shown). T cell proliferation and activation marker expression was dependent on CD86 as demonstrated by Ab blockade of this pathway (Fig. 5B, 5C).

### IL-21–mediated CD86 upregulation has functional consequences in vivo

**FIGURE 6.**
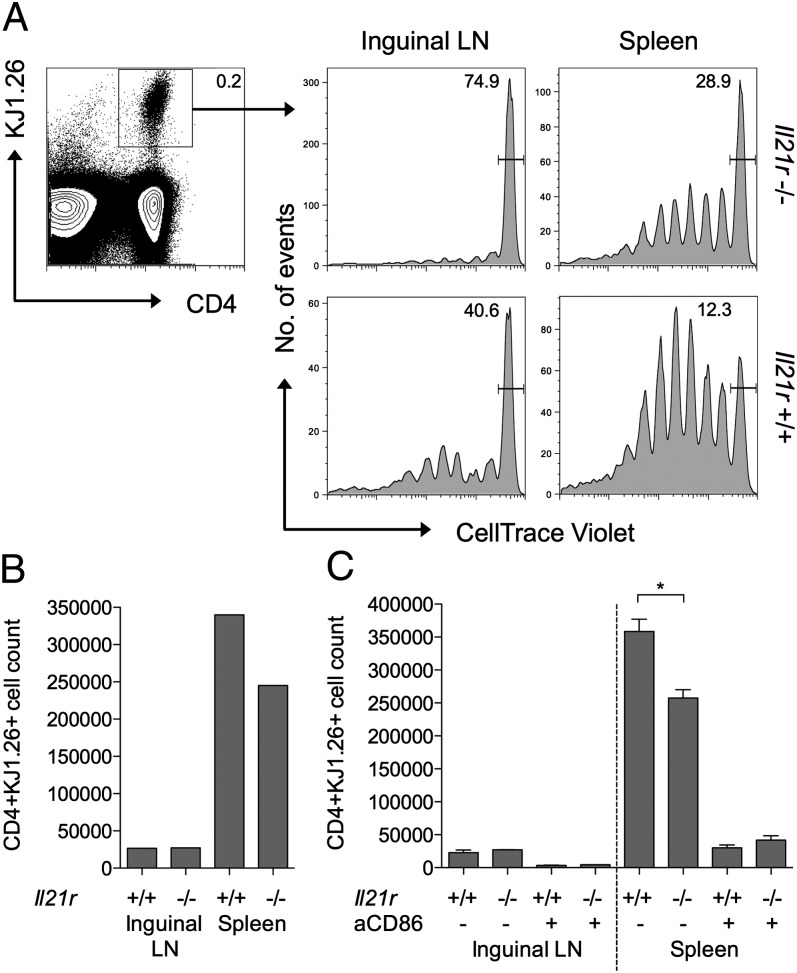
IL-21 signaling to B cells promotes CD86-dependent T cell proliferation in vivo. (**A**) OVA peptide-loaded IL-21R^−/−^ or IL-21R^+/+^ CD19^+^ B cells (3 × 10^6^) were adoptively transferred into IL-21R^−/−^ recipients. After 24 h, mice received 2 × 10^6^ CellTrace-labeled IL-21R^−/−^ DO11^+^ CD4^+^ T cells i.v. and 1 μg IL-21 i.p. Histograms show representative CellTrace dilution for gated CD4^+^DO11^+^ T cells harvested from spleens or inguinal lymph nodes of IL-21R^−/−^ or IL-21R^+/+^ B cell recipients at day 7. (**B**) Graph shows representative absolute CD4^+^DO11^+^ T cell counts from (A). (**C**) IL-21R^−/−^ T cells were activated in the presence of OVA peptide loaded IL-21R^−/−^ or IL-21R^+/+^ CD19^+^ B cells (as above) and recipient mice received 100 μg anti-CD86 or isotype control Ab i.p. on day 1. Graphs show collated absolute CD4^+^DO11^+^ T cell counts. Data are representative of at least two independent experiments. *p < 0.05.

To extend the above observations to an in vivo setting, we established a system in which peptide-pulsed B cells were used to stimulate the proliferation of Ag-specific CD4 T cells. Wild-type or IL-21R^−/−^ B cells were pulsed with OVA_323–339_ peptide and adoptively transferred into IL-21R^−/−^ hosts. OVA-specific T cells (DO11) that were also IL-21R^−/−^ were CellTrace labeled and injected into the same hosts, so that their response to the OVA-pulsed B cells could be tracked. To maximize the effects of IL-21 on B cell phenotype, rIL-21 was injected i.p. where indicated. Ensuring that the host animals and the adoptively transferred T cells were IL-21R^−/−^ allowed us to restrict the IL-21 effects to the B cell compartment. At day 7, single-cell suspensions from inguinal lymph node and spleen were stained by flow cytometry to identify the adoptively transferred Ag-specific T cells and assess their proliferation status (Fig. 6A). CellTrace profiles for gated Ag-specific T cells revealed greater proliferation in mice that had received IL-21R^+/+^ B cells compared with those that had received IL-21R^−/−^ B cells. Proliferation was particularly evident in the spleen, consistent with the likely trafficking of the adoptively transferred peptide-pulsed B cells to this site. The enhanced T cell response to IL-21R^+/+^ B cells was also reflected in higher absolute numbers of Ag-specific T cells (Fig. 6B). The ability of IL-21 signaling to B cells to augment the T cell response was dependent on CD86 as assessed by in vivo Ab blockade (Fig. 6C). Recovery of injected B cells at day 1 confirmed increased expression of CD86 on IL-21R^+/+^ B cells compared with IL-21R^−/−^ B cells (Supplemental Fig. 2). Collectively, these data demonstrate that IL-21 increases expression of CD86 on B cells and that this increased availability of costimulatory ligand has functional consequences for the magnitude of T cell responses in vitro and in vivo.

## Discussion

IL-21 is recognized to exert multiple effects on the differentiation and function of B cells. Although first recognized for its ability to promote human B cell proliferation (31), it can also trigger B cell apoptosis (32) with the outcome of IL-21R signaling most likely dictated by its context (10, 33). IL-21 has a well-established role in the induction of plasma cell differentiation (10, 11) and this involves the cooperative binding of STAT3 and IFN regulatory factor 4 to the IL-21 response element in the *Prdm1* gene encoding Blimp-1 (34). Intriguingly, IL-21 also promotes the expression of Bcl6 a key transcriptional regulator of germinal center B cell differentiation (12, 13), despite the fact that Blimp-1 and Bcl6 have mutually antagonistic effects. Thus IL-21 acts at multiple stages to modulate B cell activation, differentiation, and death. Our recent findings provide additional insight into the effects of IL-21 on B cells, demonstrating an upregulation of CD86 that has consequences for T cell–dependent immune responses.

The role of IL-21 in DCs is less well studied and our side-by-side comparison of B cells and DCs suggested CD86 was not obviously upregulated by IL-21 in the latter population. Whereas it has been shown that IL-21 can inhibit DC maturation (35, 36), other studies suggested IL-21 can enhance DC function (37), and recent analysis has identified a role for IL-21 in triggering DC migration in a virus-induced diabetes model (38). Interestingly, in the latter study, reduced migration of IL-21R^−/−^ DCs was accompanied by decreased CD86 expression. Thus, further work is required to fully elucidate the biological function of IL-21 in DCs.

The provision of CD28 costimulation is known to be of critical importance in thresholding T cell responses. As a consequence, stimuli that alter expression of the costimulatory ligands CD86 and CD80 are likely to have a significant impact on T cell immunity. This is particularly true of CD86, as it is thought to be the dominant ligand for driving T cell responses (39). Expression of CD86 and CD80 is tightly regulated, and the CD28 homolog CTLA-4 serves to limit their availability by competitive inhibition and ligand downregulation (40–42). Unregulated availability of ligands, in mice lacking CTLA-4, results in unfettered T cell responses that culminate in lethal autoimmunity (43, 44). Thus, restricting CD86 and CD80 expression represents a major control point for adaptive immunity. Counteracting the downregulation of ligand by CTLA-4, various inflammatory stimuli serve to enhance ligand expression. Accordingly, TLR ligands and cytokines such as IFN-γ have been shown to upregulate CD86 expression. In this study, we identify IL-21 as a potent upregulator of CD86 expression on B cells.

IL-21 is one of several cytokines associated with systemic autoimmunity (45) and has been linked with lupus pathogenesis in mice (10, 46) and humans (47). The capacity of IL-21 to upregulate CD86 and promote T cell costimulation could conceivably contribute to its pathogenic effects. Consistent with this, B cell expression of the costimulatory ligands CD86 and CD80 has been shown to be essential for autoreactive T cell activation and development of joint pathology in the proteoglycan-induced arthritis model in mice (48).

A major cellular source of IL-21 is the T_FH_ cell subset that provides crucial help to B cells during the germinal center reaction (49–51). Interestingly, IL-4 is also produced by T_FH_ cells and is known to positively regulate the expression of CD86 during productive interactions with B cells. Since B cell–derived CD86 provides critical signals for T_FH_ cell maintenance (52), the production of IL-21 and IL-4 provides an elegant mechanism for T_FH_ cells to solicit their own survival signals.

We show that IL-21–dependent CD86 upregulation is strictly reliant on STAT3 phosphorylation and PI3K, revealing unappreciated roles for these pathways in IL-21–mediated B cell responses. Using B cells expressing a catalytically inactive isoform of p110δ, we further identify this PI3K subunit as essential for the promotion of CD86 by IL-21. In contrast, IL-4–driven CD86 upregulation is independent of PI3K, consistent with previous studies that instead identified a role for STAT6 (53). The precise relationship between PI3K and STAT3 in orchestrating IL-21–dependent CD86 upregulation is unclear, but preliminary studies suggest that STAT3 activation is independent of the PI3K pathway (Supplemental Fig. 3). The PI3K pathway is an important mediator of lymphocyte survival (54), and inhibitors of the p110δ isoform are in clinical use for the treatment of CLL (55). Our data suggest that PI3K inhibition may have a greater impact on the nature of the immune response than previously appreciated; in addition to blocking Ag-dependent proliferation and survival signals in B cells, abrogating PI3K signaling may also influence the costimulatory profile of B cells and their ability to drive CD4 T cell responses.

IL-4 and IL-21 exhibit redundancy in their ability to support germinal center formation, with deficiency in each pathway alone having only a minor impact (9). Likewise, either IL-4 or IL-21 appears sufficient to support IgG secretion from mouse (56) or human (57) B cells in vitro with synergistic activity being seen in the presence of both. Redundant roles for IL-4 and IL-21 in B cell CD86 upregulation might explain why mice lacking the PI3K p110δ subunit in B cells retained the capacity to form germinal centers (58), because IL-4–mediated CD86 upregulation would be predicted to be intact in this setting. Although IL-21 and IL-4 are frequently coexpressed in germinal center resident T_FH_ cells (59, 60), the observation that Th17 cells can also provide cognate help to B cells (61, 62) suggests at least one scenario in which IL-21 provision may be uncoupled from that of IL-4.

Similar to IL-21, the cytokine IL-10 is well known as a plasma cell differentiation factor. Despite their largely overlapping roles in promoting plasma cell differentiation, these cytokines have notably distinct effects on CD86 expression, with IL-10 being generally accepted to downregulate CD86. Intriguingly, the receptors for these cytokines appear to be subject to differential kinetic regulation during B cell differentiation, with IL-21 playing an earlier role than IL-10 (63). This might imply a set window during which CD86 expression is amenable to IL-21–directed upregulation. If late IL-10 signals downregulate CD86, this could suggest a requirement for appropriate curtailment of T cell costimulatory signals for optimal T cell/B cell collaboration. In keeping with the idea of active downregulation (as well as upregulation) of costimulatory ligands, Bcl6 is known to downregulate CD80 in germinal center B cells (64). The fine tuning of CD86 and CD80 expression in germinal center B cells and the consequences for T_FH_ cell homeostasis (52, 65) are incompletely understood and represent key areas for further investigation.

In summary, we have shown that IL-21 upregulates CD86 expression on B cells in a manner that depends on the PI3K p110δ subunit. This finding is likely to have important implications for T cell/B cell collaboration within the germinal center, and for T cell responses in the wider context of antitumor responses and autoimmunity.
